# Plant species dispersed by Galapagos tortoises surf the wave of habitat suitability under anthropogenic climate change

**DOI:** 10.1371/journal.pone.0181333

**Published:** 2017-07-20

**Authors:** Diego Ellis-Soto, Stephen Blake, Alaaeldin Soultan, Anne Guézou, Fredy Cabrera, Stefan Lötters

**Affiliations:** 1 Department of Migration and Immuno-Ecology, Max Planck Institute for Ornithology, Radolfzell, Germany; 2 Department of Biology, University of Konstanz, Konstanz, Germany; 3 Biogeography, Trier University, Trier, Germany; 4 Whitney R. Harris World Ecology Center, University of Missouri-St. Louis, St. Louis, Missouri, United States of America; 5 WildCare Institute, St. Louis Zoo, St. Louis, Missouri, United States of America; 6 Department of Biology, Washington University, St. Louis, Missouri, United States of America; 7 State University of New York College of Environmental Science and Forestry, Syracuse, New York, United States of America; 8 Charles Darwin Foundation, Puerto Ayora, Santa Cruz, Galapagos, Ecuador; Indian Institute of Science, INDIA

## Abstract

Native biodiversity on the Galapagos Archipelago is severely threatened by invasive alien species. On Santa Cruz Island, the abundance of introduced plant species is low in the arid lowlands of the Galapagos National Park, but increases with elevation into unprotected humid highlands. Two common alien plant species, guava (*Psidium guajava*) and passion fruit (*Passiflora edulis*) occur at higher elevations yet their seeds are dispersed into the lowlands by migrating Galapagos tortoises (*Chelonoidis* spp.). Tortoises transport large quantities of seeds over long distances into environments in which they have little or no chance of germination and survival under current climate conditions. However, climate change is projected to modify environmental conditions on Galapagos with unknown consequences for the distribution of native and introduced biodiversity. We quantified seed dispersal of guava and passion fruit in tortoise dung piles and the distribution of adult plants along two elevation gradients on Santa Cruz to assess current levels of ‘wasted’ seed dispersal. We computed species distribution models for both taxa under current and predicted future climate conditions. Assuming that tortoise migratory behaviour continues, current levels of “wasted” seed dispersal in lowlands were projected to decline dramatically in the future for guava but not for passion fruit. Tortoises will facilitate rapid range expansion for guava into lowland areas within the Galapagos National Park where this species is currently absent. Coupled with putative reduction in arid habitat for native species caused by climate change, tortoise driven guava invasion will pose a serious threat to local plant communities.

## Introduction

Alien species are among the greatest threats facing global biodiversity [1, 2]. When invasive, these species can cause huge declines in the abundance and distribution of native species and dramatically modify community ecology and ecosystem function [3, 4, 5]. The ecological impacts of invasive species may be particularly detrimental to biodiversity on oceanic islands [6, 7, 8, 9] because these ecosystems often harbor a high proportion of endemic taxa of which many are endangered and where population sizes are naturally small [10, 11]. In many cases, anthropogenic climate change may increase the competitive advantage of invasive species over natives, exacerbating their negative impacts [12, 13]. These impacts may include increases or shifts in potential geographic range, as has been proposed for various invasive plants under different future climate scenarios [14, 15, 16, 17].

Native biodiversity of the Galapagos Archipelago, where endemism is high [18], is under serious and increasing threat from invasive alien species [19, 20]. A total of 870 introduced plant species are now found on Galapagos which outnumber native species by 1.6:1 [21]. Alien plants are most abundant in humid highlands, particularly in farmland [22], but decline in the drier lowlands, which prevail over much of the Galapagos National Park [23, 21]. According to Trueman et al. [24], alien species invasions on Galapagos will increase as human activities grow and because climate change will likely favour invasive over native species.

Important steps in predicting how plants spread across landscapes under climate change are first, quantifying seed dispersal characteristics, and second estimating the survival probability of seeds and seedlings [25]. Galapagos tortoises (*Chelonoidis* spp.) are prodigious long distance seed dispersers with a mean dispersal distance of 245m [26]. On Santa Cruz Island tortoises disperse seeds from at least 64 plant species, of which 27 are introduced species. Some 20% of seeds ingested by tortoises are dispersed > 1 km. Thus, long distance seed dispersal by tortoises is not a rare event and may confer considerable advantages for tortoise-dispersed plant species [27, 28].

On Santa Cruz Island, most long distance movements by tortoises usually occur during seasonal migrations which take them from hot, arid conditions near sea level to more humid, cool conditions up to ca. 400 m in elevation and back [29]. Because of the wide variety of environments traversed by tortoises during their migrations, it is likely that a portion of the seed dispersal services performed by tortoises is ‘wasted’; that is, tortoises transport seeds into environments in which they have little or no chance of germination and survival under current climate conditions.

The proportion of tortoise seed dispersal wasted under current conditions may change under future climate change. Scenarios for Galapagos predict an increase in the frequency and intensity of El Niño events and increases in hot season temperature and rainfall leading to generally more humid conditions [30]. Increased moisture can be expected to favour the spread of introduced species into the currently relatively arid lowlands, at present dominated by native species. If tortoises maintain long distance migrations under climate change, the wasted seed dispersal of today may, in the future, result in an opportunity for rapid range expansion of tortoise-dispersed plant taxa, including invasive alien species.

Here we consider this possibility for two candidate species, *Psidium guajava* L. and *Passiflora edulis* Sims. *Psidium guajava* (guava) is a small tree, which was introduced to Galapagos by local farmers in ca. 1910 for domestic use as a food source. It has spread rapidly in humid areas and is now naturalised on four islands [31]. Trees bear abundant small succulent drupes containing hundreds of seeds in a sugary pulp [32]. *Passiflora edulis* (passion fruit) was also brought to Galapagos as a domestic food source that quickly escaped and spread widely [33]. Passion fruit is an herbaceous climbing vine that also produces an abundance of seeds in a thick pulp, ideally suited to animal dispersal. Guava has been introduced around the world as both a cash crop and a smaller scale food source [34]. Similarly, passion fruit has been introduced to numerous tropical islands as a fruit source and is sometimes produced commercially [35], The mechanism by which these species arrived in Galapagos is not known, but both species were likely initially grown from seed on private land. Local consumption is now high and the fruits of both are sold commercially in local markets (Blake pers. obs.). Viable seeds from both species are dispersed by a variety of frugivores [36] including tortoises [26], mocking birds [37], cattle (SB, pers. obs.) and humans, and both species pose serious problems to the floras of numerous oceanic islands and archipelagos worldwide [38].

Our aims were to (1) estimate current and future habitat suitability for guava and passion fruit based on the current climate and on future climate predictions for 2050 and 2070 (2) quantify rates of wasted dispersal by tortoises for these species and (3) assess the role of tortoises in promoting the spread of these species now and in the future, under the assumption, justified later in the paper, that tortoise migratory behaviour will continue over the temporal scale of our climate change predictions.

## Methods

### Study area

The Galapagos Islands straddle the equator in the eastern Pacific ca. 1,000 km west of Ecuador. This volcanic archipelago includes 13 large islands (>1 km^2^), the oldest of which are ca. 4 million years old [39]. The climate is characterized by a hot wet season from January to May, and a cool dry season for the rest of the year [30]. However, during the ‘dry’ season, persistent cloud cover results in humid upland conditions on the windward (southern) slopes of the islands [40]. Tortoises once apparently occurred on nine islands, but due to anthropogenic extinctions are now found on just six [41]. Vegetation patterns are driven by rainfall and substrate which are largely determined by aspect and elevation [33].

**Fig 1:**
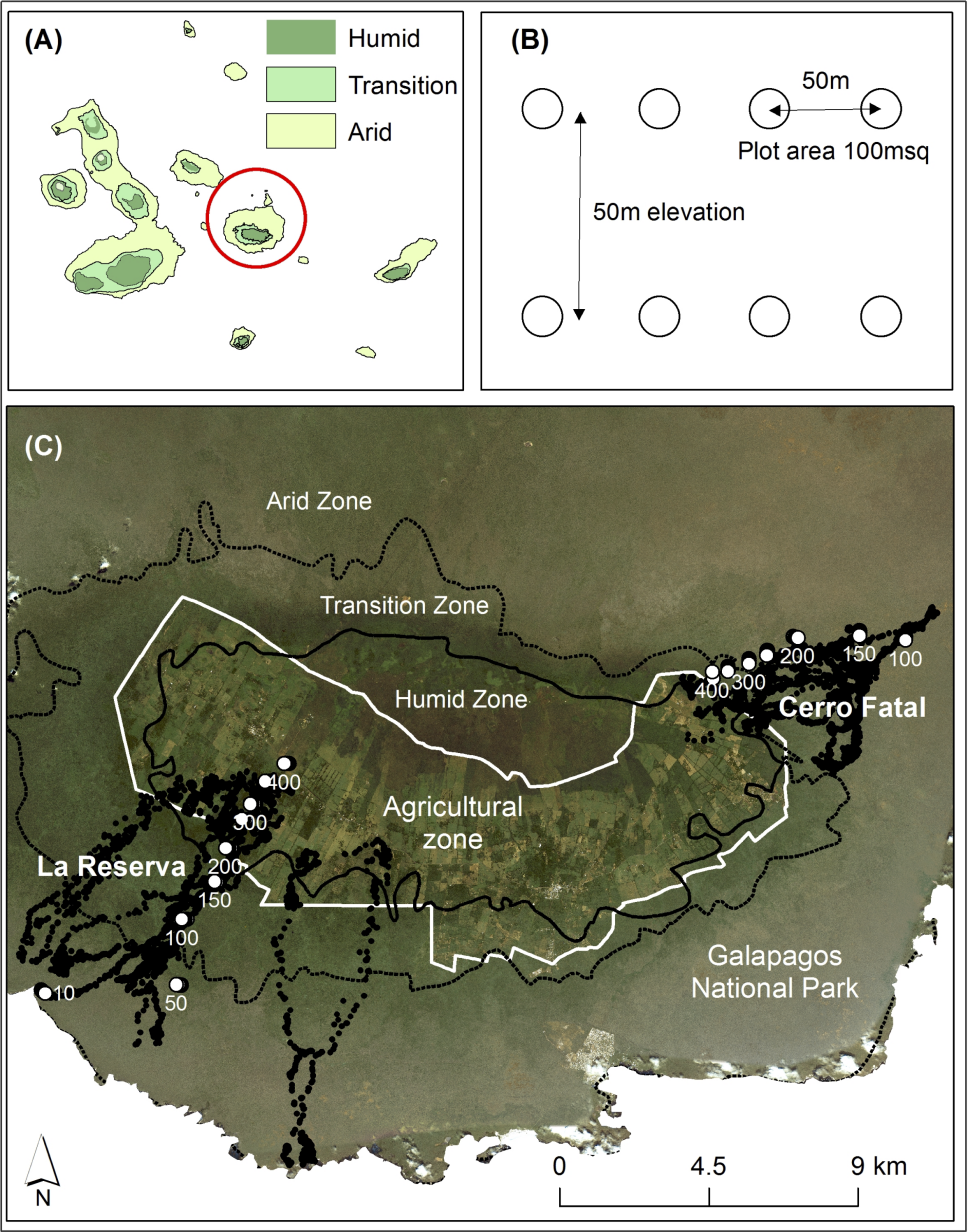
Study site. (a) The location of Santa Cruz Island in the centre of the Galapagos Archipelago, (b) field-based sample design for vegetation plots studied on Santa Cruz, and (c) major habitats and land use types and vegetation plots along two elevation gradients (La Reserva, and Cerro Fatal) and tracks of GPS-tagged tortoises (black dots)

Our study was carried out on Santa Cruz Island (Fig 1) which rises to 860 m above sea level and has an area of 986 km^2^ [42]. Five main natural vegetation zones are recognized on Santa Cruz. The coastal zone, characterized by salt-resistant plants on sandy beaches, lava and mangroves, is followed by the arid zone, dominated by xerophytic trees, shrubs and cacti on a mostly lava substrate. With increasing elevation comes the transition zone where soil and understory vegetation are more developed. The moist zone contains well-developed soil with vegetation characterized by an abundance of shrubs, herbs, ferns, and trees. Finally, the highland zone is dominated by ferns, sedges and grasses with few trees [33]. On Santa Cruz, and throughout the archipelago, the arid zone covers the greatest surface area of any vegetation type within the national park.

Santa Cruz harbours two species of tortoise. *Chelonoidis porteri* (Rothschild) occurs in an area called “La Reserva” (Fig 1C) to the south and southwest of the island. The recently described *C*. *donfaustoi* ([43], Russello, Geist and Caccone) is found in the “Cerro Fatal” region on the island’s eastern flank (Fig 1C) where it is imperilled with extinction [44]. The island also holds the largest human population on the Galapagos, estimated at > 15,000 in 2010 [45]. Human occupation has resulted in the conversion of most of the moist and highland zones to agriculture and at least 86% of these zones are now degraded by either agriculture or invasive species [46].

### Estimating the potential distributions of guava and passion fruit under current and future climates

We used correlative Species Distribution Models (SDMs) to estimate the current potential distributions of guava and passion fruit on Santa Cruz. We did so by applying a modelling approach in which both worldwide native and invasive presence records to assess the potential distributions of these species. Georeferenced species presence records were obtained from the Global Biodiversity Information Facility, GBIF (http://data.gbif.org/, accessed 17 February 2015), and processed using DIVA-GIS 7.5.0 (http://www.diva-gis.org, accessed on the 28 February 2015 of [47]. This was performed by removing locations with clearly erroneous coordinates, such as points occurring in seas or oceans, extreme elevations, and occurrences collected from herbariums. This resulted in a final dataset of 1,621 and 1,435 georeferenced presence records for guava and passion fruit respectively, ranging between 32° N– 40° S, and 155° W—180° E. We then modelled the global distribution of our plant species in their respective native and introduced range at this reduced spatial extent. To modelling current climate, we used 19 ‘bioclim’ predictors (S1 Table) for the period 1950–2000 from the WorldClim database (http://www.worldclim.org, accessed 28 February 2015) at a grid resolution of 30 arc-seconds, i.e. ca. 1 x 1 km [48]. These WorldClim climatic values describe climate independent from latitude [49], which makes them biologically useful predictors in correlative modelling and explains why they are widely used in SDMs [50].

Maxent 3.3.3k [51] was employed for SDM building (www.cs.princeton.edu/~schapire/maxent, accessed 3 March 2015). This presence-only method operates with a machine-learning algorithm following the principle of maximum entropy. It makes predictions on a taxon’s potential geographic distribution taking environmental data from geo-referenced species records and random background data [51] Maxent is a widely used SDM tool and, when used correctly [52] often provides more robust results compared to other similar methods [53] although there may be different approaches in identifying habitat suitability gradients [54]. For each species, an average of 100 global model replicates obtained via bootstrapping was generated under default settings with 10,000 random background points [51, 55]. Background data was sampled randomly for each species across the study area, taking the records as a baseline and Maxent’s logistic format (ranging 0–1) was chosen for output. Thirty percent of all species records were each randomly set aside for model testing (while all others were used for training) by estimation of the AUC–that is the Area Under the Receiver Operating Characteristic Curve [51]. Following the classification of Swets [56] and Araújo [57], AUC values range between 0.5 for models with no predictive ability and 1.0 for models giving perfect predictions, and values > 0.9 describing ‘very good’, > 0.8 ‘good’, > 0.7 ‘useable’

To avoid multicollinearity of predictors in ecological space, which could influence the model quality [58], we reduced the number of bioclim predictors in each species to five (out of 19). For this purpose, before computing models, a preliminary Maxent analysis was run using all 19 variables and applying a jack-knifing approach [59]. In this way, the most ‘stand-alone’ informative predictors were identified [51] which included the following predictors as suitable for modelling: for guava, bio4 (temperature seasonality), bio7 (temperature annual range), bio14 (precipitation of the driest month), bio16 (precipitation of the wettest quarter), bio18 (precipitation of the warmest quarter); for passion fruit, bio5 (maximum temperature of the warmest month), bio7, bio14, bio18, bio19 (precipitation of the coldest quarter).

Maxent was also used to project current climate SDMs into anthropogenic future climate change scenarios (cf. [60]). Of the various datasets available, we chose each of two scenarios for the years 2050 (He45bi50, cc45bi50) and 2070 (He45bi70, cc45bi70), based on projections of the Fourth Assessment Report of the Intergovernmental Panel on Climate Change, IPCC [61]. These were Hadley and CCSM4 (NCAR Climate System Model 01.04) scenarios. Both assume socioeconomic development and environmental consciousness. Both Hadley and CCSM, as employed here, were created using a third generation Representative Concentration Pathway (RCP) of 4.5, predicting a mean global temperature increase of 1.4°C for the period 2046–2065 and a 1.8°C for the period 2081–2100 [62, 63]. Additionally, Hadley (earth system) proves to be powerful for simulations in the tropics and for predicting vegetation dynamics [64]. Anthropogenic future climate change scenarios were obtained at resolution 30 arc seconds from the WorldClim database. DIVA-GIS was used for mapping the Maxent output, i.e. species’ potential distributions within our study area.

### Quantifying wasted seed dispersal

We defined evidence for wasted seed dispersal in terms of the difference in the geographical distribution of living plants of each species compared with the distribution of seeds of these species in tortoise dung piles. If the elevation range of seeds in tortoise dung piles exceeded that of plants, we considered this as evidence that seeds are being delivered into sites in which they are unable to germinate and survive. Evidence for wasted seed dispersal was also inferred when dung piles containing seeds were found in areas outside the respective species’ ‘survival limits’, as revealed in the modelling process (see below).

We quantified the distribution of seeds in tortoise dung piles by collecting fresh dung piles on repeated linear surveys conducted on foot that traversed the elevation gradient within the range of each tortoise species (Fig 1). Dung samples were collected during three different time periods over five years (June 2009 to November 2009, July 2011 to May 2012 and April to September 2013). Surveys were repeated every one to four days by the same observers (SB, FC, DES). In an attempt to widely sample throughout the tortoise ranges and to also avoid visually biasing our sample (i.e. preferentially collecting piles containing many seeds), we collected the first dung pile encountered each time we crossed an elevation level in multiples of 50 m (i.e. 50 m, 100 m, 150 m). This approach made it unlikely that we collected more than one dung pile from the same individual. One-hundred and fifty-nine dung piles were collected in La Reserva (LR) and 63 in Cerro Fatal (CF). All dung piles were georeferenced and their elevation was obtained from an overlaid digital elevation model (Shuttle Radar Topography Mission, https://lta.cr.usgs.gov/SRTM, accessed 21 July 2015). The number of seeds of each species was counted for each dung pile by washing dung piles with rainwater and using standard soil sieves with a final mesh of 0.5 mm. Seeds from both guava and passion fruit passing through tortoise guts are mostly viable and tortoise ingestion does not seem to influence seed germination success [26].

In 2010, we quantified the abundance of both guava and passion fruit plants along the elevation gradient within the range of each tortoise species from a sample of 64 × 50m^2^ plots. A series of four nested plots was placed 50m apart and perpendicular to the linear survey routes every 50m of elevation from 10–400m above sea level in LR and from 100 to 400 m above mean sea level in CF (Fig 1B). The presence and absence of guava and passion fruit was recorded. A total of 28 and 36 vegetation plots were sampled at sites CF and LR, respectively.

### Statistical analyses

From a total of 222 dung piles, we recorded presence of guava and passion fruit in 96 and 79 dung piles, respectively. Guava was found in 25 vegetation plots while passion fruit was found in 15 plots. We used Binomial Generalized Linear Models (GLM) to predict the presence both of guava and passion fruit plants along the current climate suitability gradient obtained from our SDMs for each species. In order to determine whether these logistic models fit the observed data in our vegetation plots, we calculated Hosmer Lemeshow goodness of fit test [65]. To determine if plant distribution differed significantly between current and future climatic conditions, we estimated the relative number of grid cells in which suitable habitat was lost and gained using the BIOMOD_RangeSize function [66] following methods of Thuiler et al.[67, 68]. We did so by summing up all grid cells in which current suitable habitat became unsuitable under future climatic conditions and by adding up all grid cells that are unsuitable under current climate conditions but become suitable under future climates. We conducted all analysis with R 3.2.0 [69] with additional packages ggplot2 [70], dismo [71], MASS [72] and Biomod2[66].

## Results

### The potential distribution of guava and passion fruit under current and future climate scenarios

**Fig 2:**
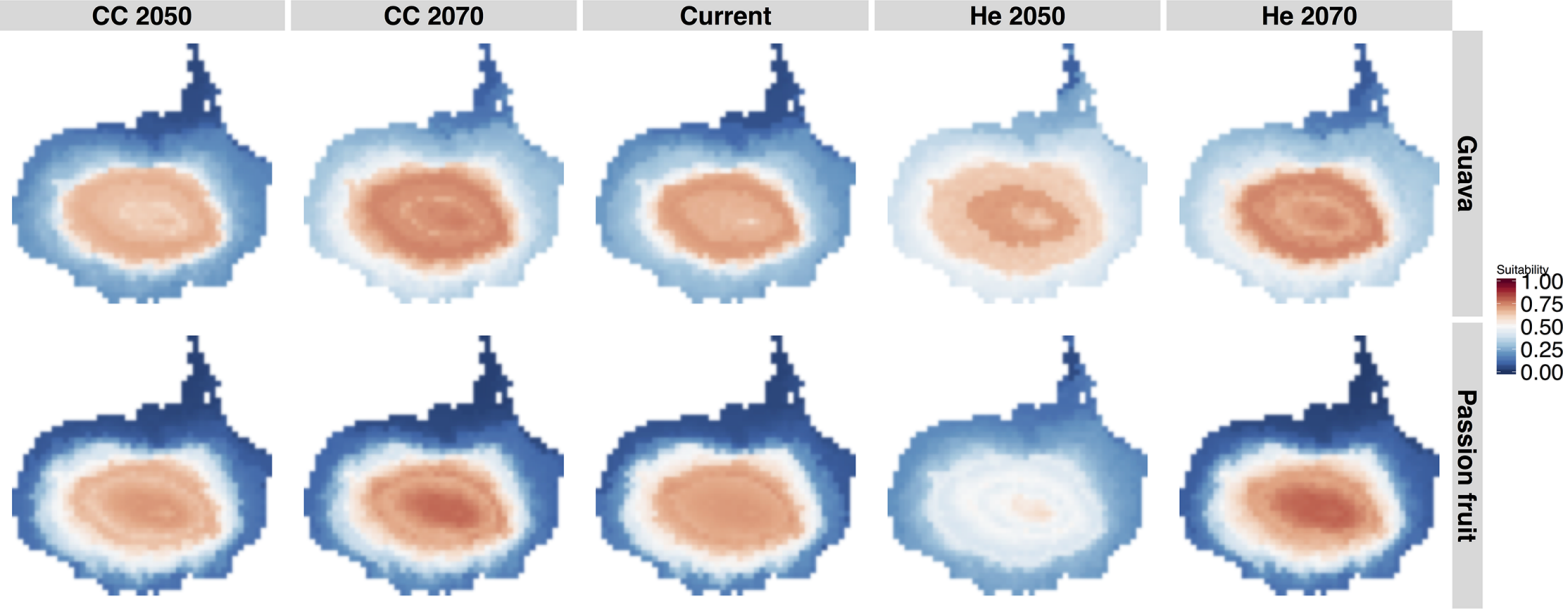
Potential distribution of guava and passion fruit on Santa Cruz Island. Derived from Maxent SDMs under current climate conditions, followed by future climate change scenarios for the years 2050 and 2070 (see text for details). Colours represent climatic suitability for the focal species. Dark red indicates higher climatic suitability and dark blue displays low suitability values.

The AUC values in the SDMs for each species exceeded 0.89, indicating that models were ‘very good’ [56]. The standard deviation for current climate projection was 0.007 and 0.005 for guava and passion fruit, respectively. Suitability for guava under current climate conditions was lowest at low elevations, rising to highest at intermediate elevations before declining to moderate values at higher elevations (Fig 2). The Hedley scenario for 2050 predicted a strong increase in suitability in lowlands, a decline at intermediate elevations and an increase at the highest elevations (Fig 2). The CCSM4 scenario for 2050 predicted little change in areas of currently low suitability, but a strong decline in areas of currently highest suitability, shifting the mid and high elevations to a more homogeneous intermediate suitability. For 2070, both climate change scenarios predicted similar outcomes with island-wide increases in suitability, with both uplands and lowlands improving in quality (Fig 2). Our quantitative assessment following methods of Thuiller et al. 2005, 2011 predicted gains in suitability in about 60% of Santa Cruz Island with no significant shrinking for 2070 (S2 Table).

In the case of passion fruit, current suitability was strongly correlated with elevation, with lowest suitability in the arid lowlands (Fig 2). Both the Hedley and CCSM4 models suggested that suitability will decrease at intermediate elevations and in the highlands by 2050; however, the Hedley model showed considerably greater declines in island-wide suitability than the CCSM4 (Fig 2). For the year 2070, both CCSM4 and Hedley models suggested an increase in areas of high suitability in the highlands, little change from current suitability in the lowlands (Fig 2) and slight overall losses of suitability (S2 Table).

### Observed distribution of guava and passion fruit plants

**Fig 3:**
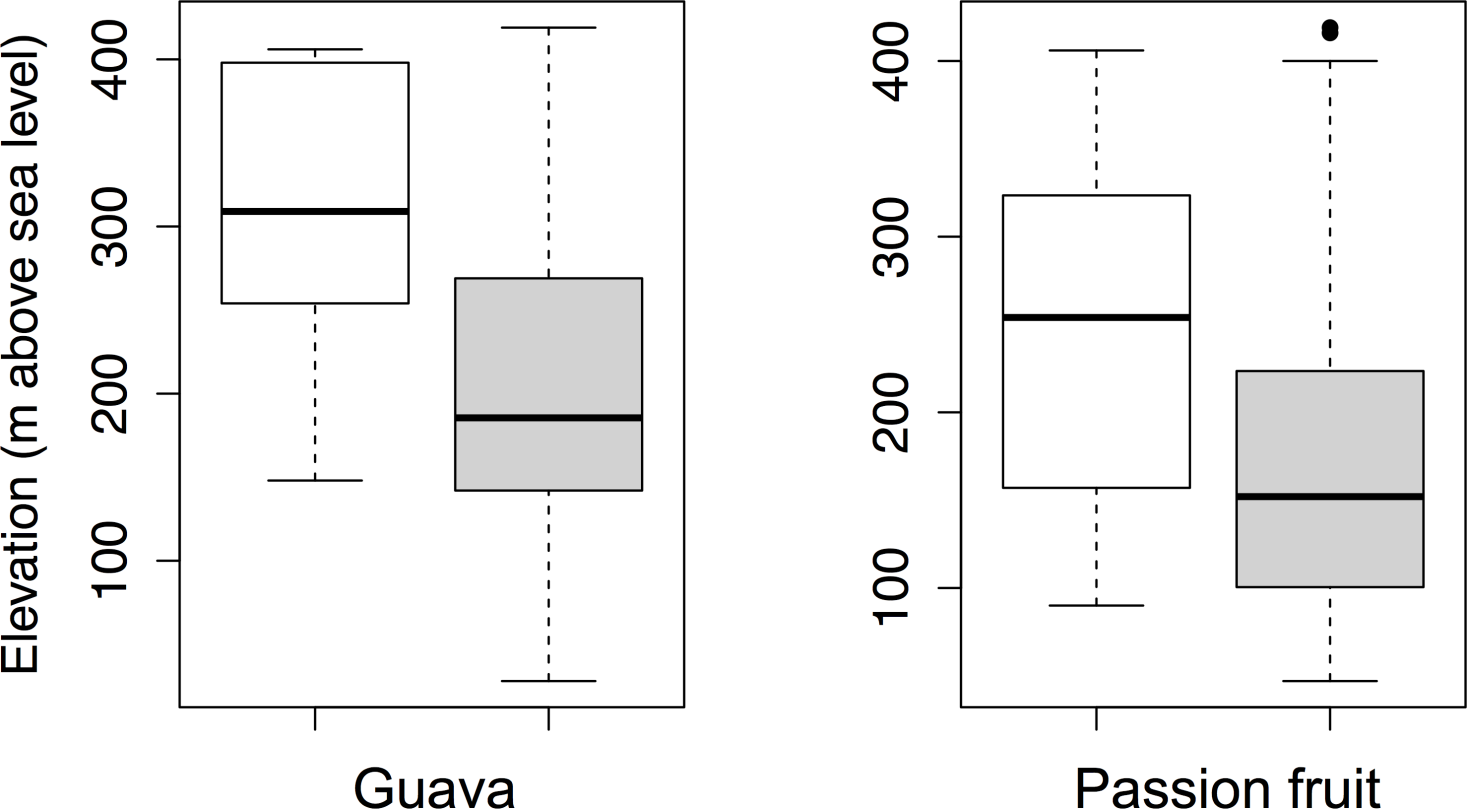
The distribution of guava and passion fruit plants in vegetation plots (white) and seeds in dung piles (grey) along the elevation gradient on Santa Cruz Island, Galapagos. Values presented fall within the inter quartile range ranging from 25^th^ to 75 percentile.

**Fig 4:**
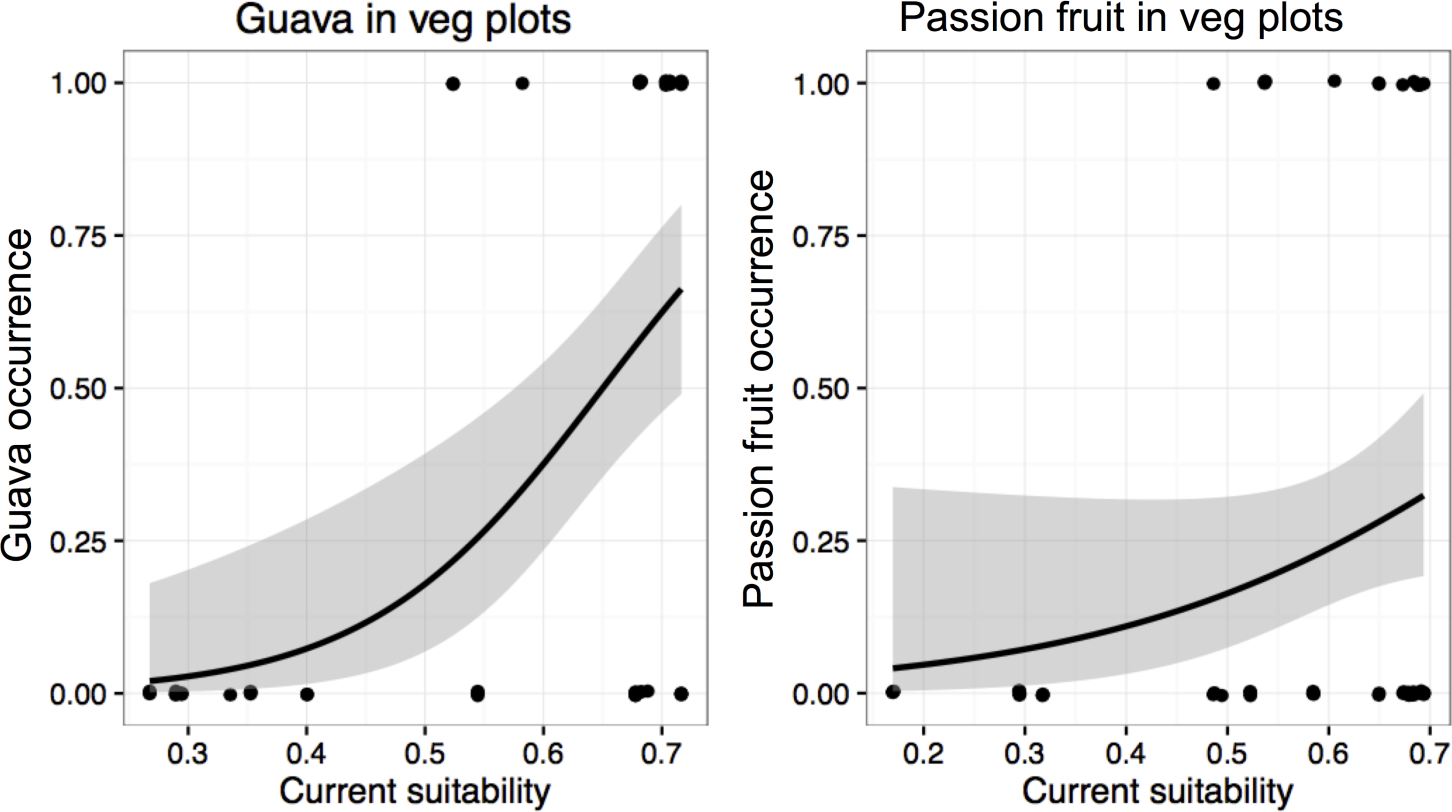
The probability of presence of guava and passion fruit plants predicted by current climatic suitability. Plant species occurrence was strongly correlated with habitat suitability for guava, but less so for passion fruit.

Guava trees were found in 21 out of 36 vegetation plots in LR and in four of 28 in CF. Passion fruit was recorded in 14 plots in LR and in just one plot in CF. The lower elevation limit were 148m and 90m, for guava and passion fruit, respectively, while the upper limits for each species exceeded our sampling range (Table 1, Fig 3). Occurrences of plants from both species in vegetation plots were always in areas of high predicted suitability under current climate conditions (Maxent suitability values > 0.5 and > 0.48 for guava and passion fruit, respectively; Table 1). All vegetation plots with predicted suitability below this ‘survival limit’ did not contain any passion fruit and guava plants (Fig 4). Furthermore, current habitat suitability was a strong predictor of plant occurrences for both guava (p < 0.01) and passion fruit (p<0.08). In both cases, a Hosmer Lemeshow goodness of fit test did not detect significant differences between the logistic model and the observed data (guava Chi-squared = 14.09, df = 8, p-value = 0.08; passion fruit Chi-squared = 12.51, df = 8, p-value = 0.13).

**Table 1:** Occurrences of plants in plots and seeds in dung piles of guava and passion fruit across elevation and suitability gradients in La Reserva (LR) and Cerro Fatal (CF) regions of Santa Cruz Island, Galapagos.

	Species	Site	Presence (%)	Elevation (m)			Suitability (Maxent value)		
				Min.	Mean	Max.	Min.	Mean	Max.
**Plot**	Guava	LR	58	148	284	406	0.524	0.668	0.716
**Plot**	Guava	CF	14	398	399	400	0.716	0.716	0.716
**Plot**	Passion fruit	LR	38	90	237	406	0.487	0.629	0.691
**Plot**	Passion fruit	CF	3	398	398	398	0.694	0.694	0.694
**Dung piles**	Guava	LR	45	28	172	416	0.267	0.546	0.716
**Dung piles**	Guava	CF	40	155	280	419	0.441	0.636	0.716
**Dung piles**	Passion fruit	LR	42	47	150	419	0.264	0.510	0.692
**Dung piles**	Passion fruit	CF	19	233	344	419	0.636	0.682	0.705

A total 36 and 28 vegetation plots and 159 and 63 dung piles were sampled at LR and CF, respectively. Minimum and maximum are abbreviated.

### Observed distribution of seeds and wasted seed dispersal by Galapagos tortoises under current conditions and future climate scenarios

Guava seeds were found in 71 dung piles (44.7%) in LR and in 25 (39.7%) in CF, while passion fruit occurred in 67 dung piles in LR (42.14%) and in 12 (19.04%) in CF. Tortoises dispersed prodigious quantities of seeds from both plant species (Table 1). A total of 138,529 guava seeds and 19,420 passion fruit seeds were recorded from 222 dung piles (guava: mean = 1,443.01 ± 2,056.81, maximum 8,726; passion fruit: mean 245.82 ± 510.92, maximum 3,721). Seeds in dung piles of both guava and passion fruit were distributed over considerably larger ranges of elevations and Maxent suitability values than were living plants (Table 1, Fig 3). Large numbers of dung piles found in areas below the current species survival limits contained seeds from both species (16.6% of all dung piles containing guava seeds and 13.1% containing passion fruit seeds). These dung piles and the seeds they contain are considered as wasted seed dispersal events. In the case of guava, some 10.6% of all seeds dispersed by tortoises were wasted. Under our assumption that Galapagos tortoise migratory behaviour remains unchanged (discussed below), in both future climate change scenarios (Hedley, CCSM4) our results suggested a decline in wasted dispersal to ca. 4% in 2050 and 2070 (Table 2). For passion fruit, ca. 30% of seeds dispersed were wasted under current climate conditions, which we predict will rise to > 45% and > 58% under the CCSM4 and Hedley scenarios respectively, before declining back to approximately current levels by 2070 (Table 2).

**Table 2:** The magnitude of wasted seed dispersal of guava and passion fruit by Galapagos tortoises under current climate conditions and two future climate change scenarios each for 2050 and 2070 on Santa Cruz Island, Galapagos.

	Species	Dung piles found below survival threshold of plants		Wasted seeds
		Number of piles	Percentage of piles	Percentage of seeds
**Current**	Guava	37	38.5	10.6
**CCSM4 2050**	Guava	27	28.1	4.4
**Hedley 2050**	Guava	17	17.7	3.2
**CCSM4 2070**	Guava	22	21.0	3.9
**Hedley 2070**	Guava	22	21.0	3.9
**Current**	Passion fruit	29	36.7	29.3
**CCSM4 2050**	Passion fruit	46	58.2	45.6
**Hedley 2050**	Passion fruit	59	74.7	58.2
**CCSM4 2070**	Passion fruit	33	41.8	27.8
**Hedley 2070**	Passion fruit	27	34.2	24.8

## Discussion

### The distribution of guava and passion fruit and wasted seed dispersal under current climate conditions

Both passion fruit and guava are invasive alien species which have already significantly altered the composition of natural ecosystems on the Galapagos Islands [33, 38, 73]. Although various animal species, such as Galapagos tortoises, distribute viable seeds of both plant taxa over large distances [26, 36], they have restricted distributions on Santa Cruz associated with local climactic conditions [31]. Field data on the distribution of guava occurred in areas of high climatic suitability predicted by our Maxent analysis, which was parameterized with global distribution data, indicating that guava has already reached most of its suitable range and is likely approaching equilibrium. However, this relationship was more ambiguous for passion fruit. The most important bioclimatic variables in restricting guava appear to be precipitation (bioclim 14 and bioclim 18) while for passion fruit it seems to be temperature (bioclim 5 and bioclim 7). By sampling vegetation plots and tortoise dung piles along altitudinal and species suitability gradients, we found that for both species seeds in tortoise dung piles have considerably greater geographic ranges at low elevations and low habitat suitability values than do plants. This supports our prediction that seeds of guava and passion fruit are being transported by tortoises into areas that are apparently unsuitable for germination and survival under current climate conditions, and are therefore examples of wasted seed dispersal (about 10% and 30% of all tortoise dispersed seeds of guava and passion fruit respectively). Model predictions of the potential distribution of both plants species indicated that presence is restricted to suitability values well above those at which seeds in dung piles were recorded. We observed that the maximum distance tortoises dispersed seeds into unsuitable area (i.e. dung piles collected at 28 and 47 m above sea level, Table 1) was about 6 km further down-slope from the lower limits of guava and passion fruit distribution.

### The impact of future climate change

Importantly, our analysis showed that the habitat suitability modelling methodology accurately predicted the current distribution of guava and of passion fruit, thus we were able to use two different climate change scenarios to predict the potential future distributions of guava and passion fruit in 2050 and 2070. Both climate change scenarios predicted considerable increases in habitat suitability for guava in both the lowlands and highlands of Santa Cruz. The predicted expansion into the lowlands is of particular importance, because this species is currently restricted to areas above 150m elevation with most of its distribution outside of the Galapagos National Park [21, 74] while the arid, low elevations inside the park remain free from guava. As suitable habitat creeps down the elevation gradient toward the coast over the coming years, tortoise seed dispersal will facilitate the spread of guava further into the park.

We have assumed that under future climatic conditions Galapagos tortoises will continue their seasonal migrations. We feel that this is likely because, firstly, the capacity for migratory behaviour is heritable and strong selective pressure toward sedentary behaviour is necessary if migration rates are to decline [75, 76]. Galapagos tortoise generation time (ca. 25 years) is too long to provide a mechanism for natural selection to occur over the time scale of anthropogenic climate change. Secondly, though future climate change is likely to reduce the strength of gradients in primary productivity along the elevation gradient by bringing wetter conditions to the lowlands, a gradient will nevertheless remain, and tortoises should continue to respond to the spatial and temporal variation in food quality and quantity (Yackulic et al. [77].

### The role of tortoises as seed dispersers under climate change

The combination of climate models with distribution data for plants and seeds allow us to predict that by 2070, <4% of guava seed dispersal by tortoises will be wasted (compared to 10.6% currently) due to an expanding front of suitable habitat moving downslope. A reduction of ca. 6% in wasted dispersal from current levels does not appear to be hugely ecologically relevant, however if we assume that a tortoise defecates on average once per day (S. Blake, pers. obs), that each dung pile contains an average of 624 guava seeds (this study), and that there are 4000 tortoises in the Tortoise Reserve (Galapagos National Park records), this represents an average increase of 149,761 seeds per day (54.7 million seeds per year) falling into suitable habitat by 2070 compared to current levels. Thus tortoises are planting seeds over large swathes of the Santa Cruz lowlands ready to germinate and establish as soon as the expanding wave of suitable conditions arrives. Our analysis indicates that suitability values will also increase higher up the elevation gradient of Santa Cruz above the current range of tortoises (Fig 1). It is likely that guava and passion fruit will spread upslope, however tortoises are unlikely to be the principle driver. Previous data indicate that tortoises do not currently migrate above the main road that bisects the Santa Cruz highlands at ca. 300–400m elevation. Furthermore, as conditions become wetter it is unlikely to drive tortoises further into the highlands because they should find greater food biomass at lower elevations than they do currently. The conservation implications of upslope invasion by guava and passion fruit are less severe than downward invasion because the highlands of Santa Cruz are already highly transformed habitats with high abundance of invasive species [21] and these areas are outside of the national park.

Key to assessing the role of tortoises in promoting the spread of invasive species under climate change is not only the number of viable seeds dispersed, but the distances over which tortoises disperse seeds compared to other dispersal mechanisms, and how rapidly suitable habitat is expanding. Among other dispersal possibilities, small and medium ground finches are known to move viable seeds through mandibulation, but these finches are usually seed predators, while Galapagos mockingbirds can defecate viable seeds [37]. The ranging behaviour of Darwin’s finches and mockingbirds is poorly known, however home range size is likely to be ca. 0.2 ha (from Mace and Harvey [78]) and natal dispersal distances are likely to be ≤ 100m (inferred from Sutherland et al. [79]). Seed dispersal distances by birds are usually much smaller than their home range size, thus it is probable that these species are moving seeds over a few tens of meters.

Introduced herbivores such as cattle are restricted to highland areas and are unlikely to contribute to the downslope spread of guava and passion fruit. Invasive goats, donkeys and pigs are also dispersers of these species and have the potential to move seeds over large areas, however their ranges and relatively rapid digesta retention times [80] suggest that they are unlikely to disperse seeds over large vertical ranges. Humans, who on Galapagos may consume large volumes of fruits from these species, are potential long distance dispersers [81] and may also contribute to the spread of these species.

By 2070, the area of suitable habitat for guava in the tortoise reserve on Santa Cruz will have moved downslope by an average of ca. 1,500m, advancing at a rate of 27m per year. Generation times of guava are in the order of 5–8 years [32]. Taking the eight year generation time for guava near their limits of suitability, for trees to advance downslope at the same pace as the suitability front, seeds would need to be dispersed at least 218 m per generation time. Birds as seed vectors would be unable to accomplish this except potentially during rare long distance movement events. While rare long distance dispersal events may be critical for species spread and persistence [28, 82, 83] they are by definition rare, and very few seeds would be deposited at the leading edge of suitability under bird dispersal. In contrast, Galapagos tortoises are already depositing millions of viable seeds every year along the advancing suitability front ready to take advantage of improving habitat quality under climate change, accelerating the spread of this species. The eradication of guava appears to be unfeasible under current management constrains [20], and unfortunately will become even more difficult in the future as a combination of climate change and long distance dispersal by tortoises will drive this species deeper into the Galapagos National Park.

## Supporting information

S1 Table Environmental variables used as predictors for species distribution modeling. (DOCX)

S2 Table Gain and loss in suitability across future climate change scenarios in comparison to current climate. (DOCX)

S1 File Plant species occurrences in vegetation plots, dung piles and point occurrences used for Maxent modeling. (ZIP)
