# Supplementary material for: Plant species dispersed by Galapagos tortoises surf the wave of habitat suitability under anthropogenic climate change
Source: PLoS One. 2017 Jul 20;12(7):e0181333. doi: 10.1371/journal.pone.0181333 (PMC5519159; doi:10.1371/journal.pone.0181333)
Supplement: S1 Table — (DOCX) [file pone.0181333.s001.docx]

**S1 Table. Environmental variables used as predictors for species distribution modeling**

Variables Description

| BIO1 = Annual Mean Temperature |
| --- |
| BIO2 = Mean Diurnal Range (Mean of monthly (max temp - min temp)) |
| BIO3 = Isothermality (BIO2/BIO7) (* 100) |
| BIO4 = Temperature Seasonality (standard deviation *100) |
| BIO5 = Max Temperature of Warmest Month |
| BIO6 = Min Temperature of Coldest Month |
| BIO7 = Temperature Annual Range (BIO5-BIO6) |
| BIO8 = Mean Temperature of Wettest Quarter |
| BIO9 = Mean Temperature of Driest Quarter |
| BIO10 = Mean Temperature of Warmest Quarter |
| BIO11 = Mean Temperature of Coldest Quarter |
| BIO12 = Annual Precipitation |
| BIO13 = Precipitation of Wettest Month |
| BIO14 = Precipitation of Driest Month |
| BIO15 = Precipitation Seasonality (Coefficient of Variation) |
| BIO16 = Precipitation of Wettest Quarter |
| BIO17 = Precipitation of Driest Quarter |
| BIO18 = Precipitation of Warmest Quarter |
| BIO19 = Precipitation of Coldest Quarter |

**Environmental variables used for model development of *Psidium guajava***

| Variables | Percent contribution | Permutation importance |
| --- | --- | --- |
| BIO 4 | 33.6 | 41 |
| BIO7 | 4.9 | 6 |
| BIO14 | 21.5 | 25.8 |
| BIO16 | 4.9 | 6.6 |
| BIO18 | 35.1 | 20.5 |

Variables Percent Contribution

**Environmental variables used for model development of *Passiflora edulis***

| Variables | Percent contribution | Permutation importance |
| --- | --- | --- |
| BIO 5 | 28.9 | 23.1 |
| BIO7 | 21.3 | 21.8 |
| BIO14 | 16.2 | 20.2 |
| BIO18 | 24 | 16.5 |
| BIO19 | 9.6 | 18.5 |

Variables Percent Contribution
