# Supplementary material for: Plant species dispersed by Galapagos tortoises surf the wave of habitat suitability under anthropogenic climate change
Source: PLoS One. 2017 Jul 20;12(7):e0181333. doi: 10.1371/journal.pone.0181333 (PMC5519159; doi:10.1371/journal.pone.0181333)
Supplement: S2 Table — (DOCX) [file pone.0181333.s002.docx]

**S2 Table. Gain and loss in suitability across future climate change scenarios in comparison to current climate.**

|  | **Species** | **Percentage of loss** | | **Percentage of gain** | |
| --- | --- | --- | --- | --- | --- |
| **CCSM4 2050** | Guava | 12 | 1 | |  |
| **Hedley 2050** | Guava | 0 | 77 | |  |
| **CCSM4 2070** | Guava | 0 | 58 | |  |
| **Hedley 2070** | Guava | 0 | 63 | |  |
| **CCSM4 2050** | Passion fruit | 1 | 3 | |  |
| **Hedley 2050** | Passion fruit | 14 | 0 | |  |
| **CCSM4 2070** | Passion fruit | 3 | 0 | |  |
| **Hedley 2070** | Passion fruit | 6 | 0 | |  |

Gain and loss in suitability across future climate change scenarios in comparison to current climatic suitability for guava and passion fruit respectively. Analysis was performed following methods described and performed in Thuiller et al. (2005, 2011).

**References**

Thuiller W, Lavorel S, [Araújo](http://www.pnas.org/search?author1=Miguel+B.+Ara%C3%BAjo&sortspec=date&submit=Submit) MB, Sykes MT, Prentice IC. Climate change threats to plant diversity in Europe. Proc. Natl. Acad. Sci. U. S. A. 2005; 102: 8245–8250.

Thuiller W, Lavergne S, Roquet C, Boulangeat I, Lafourcade B, Araujo MB. Consequences of climate change on the tree of life in Europe. Nature. 2011;470: 531–534.
